# A comprehensive evaluation of an ELISA for the diagnosis of the two most common ascarids in chickens using plasma or egg yolks

**DOI:** 10.1186/s13071-017-2121-9

**Published:** 2017-04-18

**Authors:** Gürbüz Daş, Mark Hennies, Birgit Sohnrey, Shayan Rahimian, Kalyakorn Wongrak, Manuel Stehr, Matthias Gauly

**Affiliations:** 1 Institute of Nutritional Physiology ‘Oskar Kellner’, Leibniz Institute for Farm Animal Biology, Wilhelm-Stahl-Allee 2, 18196 Dummerstorf, Germany; 2 TECOdevelopment GmbH, Marie-Curie-Str. 1, 53359 Rheinbach, Germany; 3 Department of Animal Sciences, University of Göttingen, Albrecht-Thaer-Weg 3, 37075 Göttingen, Germany; 4 Faculty of Agriculture and Life Science, Chandrakasem Rajabhat University, 39/1 Ratchadaphisek Road, Chatuchak, 10900 Bangkok, Thailand; 5 Free University of Bozen - Bolzano, Faculty of Science and Technology, Universitätsplatz 5, 39100 Bolzano, Italy

**Keywords:** Helminth, Nematode, Non-invasive diagnosis, Poultry, ROC analysis, Test accuracy

## Abstract

**Background:**

Classical faecal egg counts (FEC) provide less reliable diagnostic information for nematode infections in chickens. We developed an ELISA based on *Ascaridia galli* antigens and tested two hypotheses, as follows: (i) IgY antibodies developed against *A. galli* will also be useful to identify *Heterakis gallinarum* infections, and (ii) circulating antibodies stored in egg yolks are as good as plasma samples, so a non-invasive diagnosis is possible. The aim of this study, therefore, was to compare the diagnostic accuracy of the ELISA system with FEC, using both plasma and egg yolks from experimentally infected hens. In addition, naturally infected animals were evaluated to validate the assay.

**Results:**

The assay quantified large differences (*P* < 0.001) in plasma or in egg-yolk IgY concentrations between infected and uninfected animals in two experiments, each performed with either of the nematode species. The assay performed with high accuracy as quantified with the area under the ROC curve (AUC) values of > 0.90 for both nematodes using either plasma or egg yolks. Sensitivity of the assay was 94 and 93% with plasma and egg yolk samples, respectively, whereas FEC yielded in a sensitivity of 84% in *A. galli* experiment. Total test accuracy of the assay with plasma samples (AUC = 0.99) tended to be higher (*P* = 0.0630) than FEC (AUC = 0.92) for *A. galli*, while the assay with either sample matrix performed similar to FEC (AUC ≥ 0.91) for *H. gallinarum.* Among the three tests, the FECs correlated better with *A. galli* burden than the ELISA. Although 90% of naturally infected hens were correctly identified by the ELISA, 45% of the infected hens tested negative with FEC, indicating the validity of the higher test accuracy of the ELISA.

**Conclusions:**

Antigens of *A. galli* can be used successfully to identify *H. gallinarum*-infected animals, indicating that chickens develop cross-reactive antibodies against the two closely related species. Egg yolks are as informative as plasma samples, so that animal welfare-friendly sampling is possible. Although the assay with plasma samples reveals qualitative information of higher quality than FECs on the infection status of naturally infected birds, the latter is still a better tool to assess the intensity of *A. galli* but not of *H. gallinarum* infections.

**Electronic supplementary material:**

The online version of this article (doi:10.1186/s13071-017-2121-9) contains supplementary material, which is available to authorized users.

## Background

Infections of chickens with gastrointestinal nematodes, particularly with the roundworms *Ascaridia galli* and *Heterakis gallinarum,* are re-emerging in European laying hen farms that operate with the obligatory non-cage housing systems [1–5]. Although a few other nematode species, e.g. *Syngamus trachea* and *Capillaria* spp., and a few cestode species are also encountered [2], the highest prevalence and worm burdens are from two phylogenetically closely related [6, 7] nematode species, *A. galli* and *H. gallinarum*. Both species are pathogenic to chickens [8, 9] and can impair overall productivity of hens through direct and indirect effects. Direct effects result from the pathogenic consequences of infections, e.g. damage to the intestinal tissue [10] and resulting impairments in overall nutrient absorption, utilization and growth [11–13] as well as mortality, which may be caused by the obstruction of intestines in heavy infections with *A. galli* [14], although recent evidence also indicates an association between the presence of both nematode infections and hen mortality [15]. Vectoring roles for other parasites (e.g. *Histomonas meleagridis* by *H. gallinarum* [16]) or bacteria (e.g. *Salmonella enterica* by *A. galli* [17]) and the impaired humoral responses after vaccinations against other pathogens (e.g. Newcastle disease virus) [18] may be considered as the most important indirect effects of the infections. Moreover, animal welfare, which was expected to improve with the EU legislative ban on battery cages [19], is also threatened/endangered because of the overall effects of the infections on animal health and welfare [20].

The occurrence, and in some cases, the intensity of nematode infections in living chicken hosts are classically determined by faecal egg counts (FEC). However, obtaining suitable individual faecal samples from the chicken host is problematic because of the small host size, which does not allow a direct faecal sampling from the rectum. Another limiting factor is the naturally occurring diurnal fluctuations in egg excretion of chicken nematodes [21]. Thirdly, egg counting techniques with random faecal samples provide less reliable information for the detection and quantification of *H. gallinarum* infections [22] because this species is located in the caeca and its eggs are shed irregularly to the external environment through the caecal faeces only a couple of times per day [23, 24]. Thus, other diagnostic techniques are needed, particularly those that are host-friendly and non-invasive.

Immunity to nematode infections in chickens seems to be regulated primarily by the mechanisms involved in cell-mediated immunity [8, 25] as is known for mammals [26], and appears to be under strong genetic control [4, 27]. No protection by humoral immunity was observed in birds immunized with soluble *A. galli* antigens [28]. Although the presence of circulating antibodies against a nematode species does not necessarily indicate an established protective immunity [28, 29], it indicates the presence of a past or actual infection history with nematodes. Thus, it has the potential to be used as an indication of infection and is of diagnostic importance. Serological analyses have been employed to assess the humoral immune response of chickens to *A. galli* infection [8, 25, 28–30]. Only Martín-Pacho et al. [30] has used an Enzyme-Linked Immunosorbent Assay (ELISA) to identify naturally infected animals; however, in that study, the sampled hens were not examined for worm burden, the current gold standard for confirming the occurrence and intensity of nematode infection, leaving the validity of the assay undetermined. Because bleeding animals to obtain plasma or serum is invasive and requires an authorized person (e.g. a veterinarian or trained technician), biological material that can be collected in a non-invasive way is both practical and important for animal welfare. In this respect, chicken eggs may be useful, as it is known that the transfer of circulating antibodies to egg yolks occurs in chickens [25, 31], analogous to cross-placental transmission in mammals [32].

Because *A. galli* and *H. gallinarum* share the same host animal, e.g. the chicken, they co-exist in most cases of naturally occurring infections [2, 3, 5] across a wide geographic area [5] and are genetically closely related species [6, 7], it is, therefore, reasonable to assume that these two parasites may induce similar antigen-induced antibody responses in the chicken host. Consequently, antibodies raised against one species (e.g. *A. galli*) may show cross-reactivity due to the existence of the other species (e.g. *H. gallinarum*) and *vice versa*. To test whether both parasite species induce the production of cross reactive antibodies, samples for serological analyses that are derived from both mono-species- and multi-species-infected hosts are required. We developed an ELISA based on *A. galli* antigens to measure antibodies raised against both *A. galli* and *H. gallinarum* in chicken plasma and egg yolks. We then tested two hypotheses, as follows: (i) antibodies developed against *A. galli* will, beyond cross-reactivity, also be useful to identify *H. gallinarum*-infected animals, and (ii) circulating antibodies accumulated in egg yolks are as informative as plasma samples, so that non-invasive sampling may be possible. Therefore, the aim of this study was to compare the diagnostic accuracy of the ELISA system with faecal egg counts using both plasma and egg yolk samples from experimentally infected and uninfected control birds. The validation of the assay using plasma samples from naturally multi-nematode species infected animals was additionally addressed.

## Methods

### Enzyme linked immunosorbent assay (ELISA)

Plasma *A. galli-*specific antibodies were determined using antigen-coated microtiter wells. Bound antibodies were measured by an enzyme-conjugated secondary antibody against chicken IgG (IgY). A simplified schematic representation of the ELISA format is shown in Additional file 1: Figure S1a.

### Antigen isolation from *A. galli*

For antigen isolation, thawed worms were washed three times with phosphate-buffered saline (PBS), followed by one wash with 70% ethanol and another PBS wash. After homogenization in a mortar, one part of worms was extracted with two parts of a basic buffer (35 mM BisTris, 25 mM Tris, pH 9) for 5 min. After centrifugation at 17000× *g* at 4 °C for 15 min, the supernatant was collected (soluble antigens). The pellet was washed twice by re-suspending in the same buffer and centrifuging. This pellet was then suspended in a buffer containing 100 mM sodium acetate, pH 5, containing 2% SDS, 50 mM TCEP (Fisher Scientific GmbH, Schwerte, Germany), 1 mM EDTA and incubated at room temperature for 1 h before centrifugation as described above. The supernatant was collected (solubilized antigens). After protein measurement, both supernatants that contained soluble and solubilized antigens were used in a 1:1 protein ratio for coating the microtiter plates. *Ascaridia galli* proteins with potential antigenic properties in both soluble proteins as well as in the solubilized pellet are shown in the SDS-PAGE (sodium dodecyl sulfate polyacrylamide gel electrophoresis) analysis, provided in Additional file 1: Figure S1b.

During the initial steps of the assay development, antigens of *H. gallinarum* were also isolated as described for *A. galli*. Thereafter, plasma samples from birds infected either with *A. galli* or *H. gallinarum* as well as from uninfected control birds were analysed with both assay prototypes, each developed based on worm-specific antigens for the diagnosis of either *A. galli* or *H. gallinarum* (see Additional file 2: Figure S2). Because there were reasonably high correlations (*r*_(39–45)_ ≥ 0.70, *P* < 0.0001) between plasma *A. galli*- and *H. gallinarum*-specific IgY antibody concentrations quantified with either assay, and both assays differentiated satisfactorily between infected and control birds, it was decided to use the assay developed for the diagnosis of *A. galli* because it is much easier to obtain the required antigens for the assay from the larger worm *A. galli* than from *H. gallinarum*.

### Assay procedures

EIA/RIA 1 × 8 strip-well microtiter plates (Nunc AIS, Nunc PolySorp 469078) were coated overnight at 4 °C with 100 μl *A. galli* extract at a concentration of 10 μg/ml in coating buffer (0.05 M sodium dihydrogen-phosphate; 0.03% 5-Bromo-5-nitro-1.3-dioxane (BND) SDT - Stereospecific Detection Technology, Baesweiler, Germany; pH 7.4). After the overnight incubation, the wells were blocked with blocking buffer (20% PBS; 0.1% tween 20; 0.06% BND) for 1 h and then washed 5 times with 350 μl washing buffer (1:2 diluted blocking buffer) and dried at room temperature.

Plasma samples were incubated at a dilution of 1:2,500 in assay buffer (0.02 M disodium hydrogen-phosphate; 0.01 M EDTA; 0.2% BSA; 0.12 M sodium chloride; 0.005% chlorhexidine di-gluconate; 0.1% gelatine hydrolysate; 0.05% Tween 20; 0.02% phenol red; 0.06% BND) for 2 h on a plate shaker. After incubation, the plates were washed 5 times with washing buffer. Then, 100 μl of enzyme conjugate (peroxidase conjugated goat anti-chicken IgG antibodies (Rockland Immunochemicals, Limerick, PA, USA) at 100 ng/ml in an assay buffer containing 10% normal goat serum (PAN-BIOTECH, Aidenbach, Germany) was incubated for 30 min on a plate shaker. After an additional 5 washes, the TMB substrate (TMBS, SurModics, MN, USA) was added for 30 min on the plate shaker, followed by termination with 100 μl of 1 M hydrochloric acid and an OD measurement at 450 nm. Antibody binding was expressed relative to a standard chicken serum with high antibody activity (1,000 mU/ml per definition). All further sample antibody binding results were expressed in relation to the standard chicken serum, which was used serially diluted in every assay as standard curve using a 4-parameter logistic (4-PL) for calculation of unknown samples.

The inter-assay coefficient of variability (CV) was 11.7% (4 samples, 10 assays). The overall intra-assay CV (24 samples, 2 assays) was 4.7% (3.6 and 5.7% for the first and second assays, respectively).

### Experimental infections

Most of the data presented in this study originated from two independent mono-species infection experiments using the chicken host. Each experiment was performed separately with a nematode species, either *A. galli* or *H. gallinarum*. Plasma, egg yolk and faecal samples were collected from 106 white leghorn (Lohmann Selected Leghorn) chickens that survived until the end of the studies and were necropsied for worm burden from the two independent experiments. The birds had been purchased from a commercial hatchery as one-day-old birds and reared under helminth-free conditions until the experimental infections. The birds were kept under floor-husbandry conditions on wood-shavings as litter and fed *ad libitum* commercial diets to supply age-specific nutritional needs. Age of the birds at the infection was 16 and 4 weeks in *A. galli* and *H. gallinarum* experiments, respectively. Necropsies were then performed 28 or 30 weeks (wk) post-infection (p.i.) at an age of 44 or 34 weeks in the experiments with *A. galli* and *H. gallinarum*, respectively. In each experiment, the birds were kept either as uninfected controls or experimentally infected with 1,000 embryonated/infective eggs of either nematode. Numbers of birds in the uninfected control groups were 9 and 25 in *A. galli* and *H. gallinarum* experiments, respectively. Numbers of birds in the corresponding infected groups were 31 and 41, respectively.

The birds were necropsied after electrical stunning for post-mortem parasitological examinations. The small intestines and caeca were opened, and the contents were sieved through a 100 μm mesh with tap water. The residues on the sieve were transferred into Petri dishes for counting larvae and adult worms of both sexes using a stereomicroscope. Experimental infection procedures and the post-mortem parasitological examinations followed the recommendations for the experimental infection of chickens [33] and were similar to previous studies with either nematode species [11, 13, 34]. Worms were classified as larvae, male and female adult worms based on general morphology. For *H. gallinarum*, female worms were further classified as egg-containing mature females or immatures without eggs in their uteri. Average worm length was estimated by measuring randomly selected 10 male and 10 female worms (only matures for *H. gallinarum*) of each species. Larvae were not measured. The infection experiment with *H. gallinarum* excluded *Histomonas meleagridis,* a (hyper-) parasite that is vectored by *H. gallinarum* and is able to parasitize both *H. gallinarum* and the definitive host e.g. the chicken [12, 16]*.* This was achieved by using infective *H. gallinarum* eggs obtained from worms residing in birds that were treated against *H. meleagridis* starting from 2 days before and for 7 days after the oral inoculation with infective eggs of *H. gallinarum*. The details of the methodology used for the treatment with dimetridazole against *H. meleagridis* are given in a previous report [12].

### Faecal, egg yolk and plasma samples

During the final days of life, the birds were kept in individual cages for 24 h to collect daily total faeces and individual chicken eggs. The egg yolks were separated and stored at -20 °C. Starting from the infection day on, individual blood samples were collected fortnightly, e.g. every second week p.i. to monitor seroconversion. Blood was taken from the wing vein (*vena cutanea ulnaris*) into vials containing potassium-EDTA (Sarstedt AG & Co, Nümbrecht, Germany). Blood was also collected at necropsy immediately after neck bleeding following the electrical stunning. The blood samples were centrifuged at 1619× *g* for 10 min, and the plasma was stored at −20 °C until analysis.

All the fresh daily total faeces were mixed thoroughly for each individual, and a random sub-sample (4 g) was analysed with a modified McMaster egg counting technique [35] to determine egg concentration in the faeces (number of eggs per gram faeces, EPG). The minimum detection level (MDL) of the technique was 50 EPG. Further details of sample processing are described in previous studies dedicated to the quantification of egg excretion and fecundity of *H. gallinarum* [22, 34].

### Validation of the ELISA with plasma samples from the field

An additional data set was derived from a field study as described below. For validation of the ELISA with field data, we analysed plasma samples obtained from birds with naturally occurring mixed-nematode infections and compared with the FECs. The plasma samples were obtained from naturally infected hens that were used in a previous study exploring genetic resistance to nematodes [4]. The vast majority (> 99%) of the hens were infected with at least one of three most common nematodes (*A. galli*, *H. gallinarum* and *Capillaria* spp*.*). For the present study 40 hens with both faeces and plasma samples available were used. The hens originated from two genotypes of Lohmann Brown (LB; i.e. LB Classic and LB Plus) and shared highly similar genetic backgrounds, with LB Plus hens being additionally improved for a higher body size and feed intake. The faecal and plasma samples were collected from these hens at necropsy for quantification of worm burden at 79 or 88 weeks of age for the LB Classic and LB Plus genotypes, respectively. Faecal and plasma samples were examined for EPG, and the *A. galli* antibodies were measured as described for the experimentally infected birds. The hens of both genotypes were kept together on an organic farm and housed separately in two different mobile stalls. For further details of the animals, samples and infections see Wongrak et al. [3, 4].

### Statistics

Worm burden, FEC, and plasma and egg yolk antibody concentrations were transformed using a natural logarithmic (ln) function [Ln (*y*) = ln (*y* + 1)] to correct for heterogeneity of variance and to produce approximately normally distributed data. Statistical analyses were performed using the log-transformed data separately for the *A. galli* and *H. gallinarum* experiments, except for the seroconversion data (see below).

Differences in plasma and egg yolk antibody concentrations between infection groups (control *vs* experimentally infected) were analysed with one-way ANOVA using procedure GLM of SAS [36]. Control birds were excluded from the analyses for worm burden and faecal egg count data. Correlations among worm burden and faecal egg counts as well as plasma and egg yolk antibody concentrations were analysed by Pearson correlation analysis using SAS [36]. Correlations were also based on log-transformed data.

Data consisting of plasma and egg yolk antibody concentrations as well as FEC of experimentally infected birds and their fully-matched uninfected controls at necropsy were used to assess diagnostic accuracy of the assay. For this purpose a Receiver Operating Characteristic (ROC) analysis was performed to determine objective non-arbitrary cut-off values, sensitivity, specificity and area-under-the-ROC-curve (AUC) as a measure of overall test accuracy [37]. The ROC analyses with a pre-test probability of 50 and cost ratio of 1 as well as pairwise comparisons among ROC curve areas for each test (ELISA-plasma, ELISA-egg yolk and FEC) were performed using SigmaPlot 11.0 [38]. Because three different sample types (plasma, egg yolk and faeces) were obtained from the same individuals, pairwise comparisons of the ROC areas were performed using the method of DeLong, DeLong and Clarke-Pearson by selection of the paired data type option of SigmaPlot 11.0 [38]. Interpretations of AUC were based on the accuracy categories [37, 39]. Briefly, accuracy of an assay is classified based on AUC either as low (0.5 < AUC ≤ 0.7) or moderate (0.7 < AUC ≤ 0.9) or high (AUC > 0.90).

An additional set of data containing information on seroconversion time (weeks required until cut-off values are achieved for the first time) in experimentally infected chickens in two studies were also analysed with one-way-ANOVA using SAS [36]. The data set constituted single time records of each experimentally infected animal recording the first time that an individual plasma antibody concentration was above the cut-off value determined for each nematode species. The records were based on an every-second-week time interval starting from the time of infection, implying that the individual seroconversion point could be detected at any post-infection week with an even number (e.g. at 2^nd^ or 4^th^ … week) during the study period.

Log-transformed data obtained from the field study were analysed with one-way-ANOVA to compare plasma antibody concentrations, FECs as well as worm counts of two genotypes. By using a cut-off determined for experimentally *A. galli*-infected animals, true positive and false negative cases in each genotype were determined and presented graphically.

Graphical representation of all the data was performed using SigmaPlot 11.0 [38] and JMP 12 [36] software.

## Results

### Presence and intensity of infections

Except for one bird that was experimentally infected with *A. galli*, all of the infected birds harboured worms at the end of the study. This single bird was infection-positive by FECs at an earlier time point (data not shown). On average, an *A. galli*-infected bird harboured 50 ± 5.7 (Mean ± SE) worms, of which 22% were larvae (Table 1). Average female worm length was 93 mm and the proportion of female worms to male worms was in favour of females. On average, a female *A. galli* shed approximately 9,000 eggs within 24 h through 146 g faeces (Table 1).

**Table 1:** Worm counts and further parameters (Mean ± SE) describing infections of chickens with *Ascaridia galli* or *Heterakis gallinarum*

Item	Experimental infections^a^	
	*A. galli* ^b^	*H. gallinarum* ^c^
Host (*n*)	31	41
Larva (*n*/bird)	11 ± 2.8	83 ± 11.3
Male (*n*/bird)	14 ± 1.9	193 ± 15.4
Female total (*n*/bird)	25 ± 3.4	260 ± 20.6
Immature female (*n*/bird)	nd	20 ± 3.3
Mature female (*n*/bird)	nd	240 ± 18.6
Total worm burden (*n*/bird)	50 ± 5.7	536 ± 42.5
Female worm length (mm)	93 ± 1.4	9.5 ± 0.09
Daily faeces (g/bird)	146 ± 6.6	138 ± 4.6
EPG (*n*/g faeces)	1,803 ± 598	335 ± 63.3
EPD (*n*/day)	223,762 ± 69,081	46,777 ± 8,821
Fecundity (EPD/female, total)	8,928 ± 2830	218 ± 37
Fecundity (EPD/mature female)	nd	234 ± 40

^a^Only birds that survived to the end of the experiments were included in this study

^b^Birds were infected with 1,000 infective eggs of *A. galli* and necropsied 28 weeks p.i. at an age of 44 weeks

^c^Birds were infected with 1,000 infective eggs of *H. gallinarum* and necropsied 30 week p.i. at an age of 34 weeks

*Abbreviations*: *nd* not determined, *SE* standard error

Overall, the average *H. gallinarum* burden was 536 ± 42.5 worms per bird, and the larval stages accounted for 15% of the worm population (Table 1). Immature female worms constituted approximately 8 and 4% of all females and all worms, respectively. Proportion of female (mature + immature) to male worms was in favour of females. Faecal egg concentration was on average 335 ± 63.3 EPG and each animal excreted daily approximately 138 g faeces, which then represented a fecundity estimate of 234 eggs per mature female worm within 24 h.

### Plasma and egg-yolk IgY antibody

**Fig. 1:**
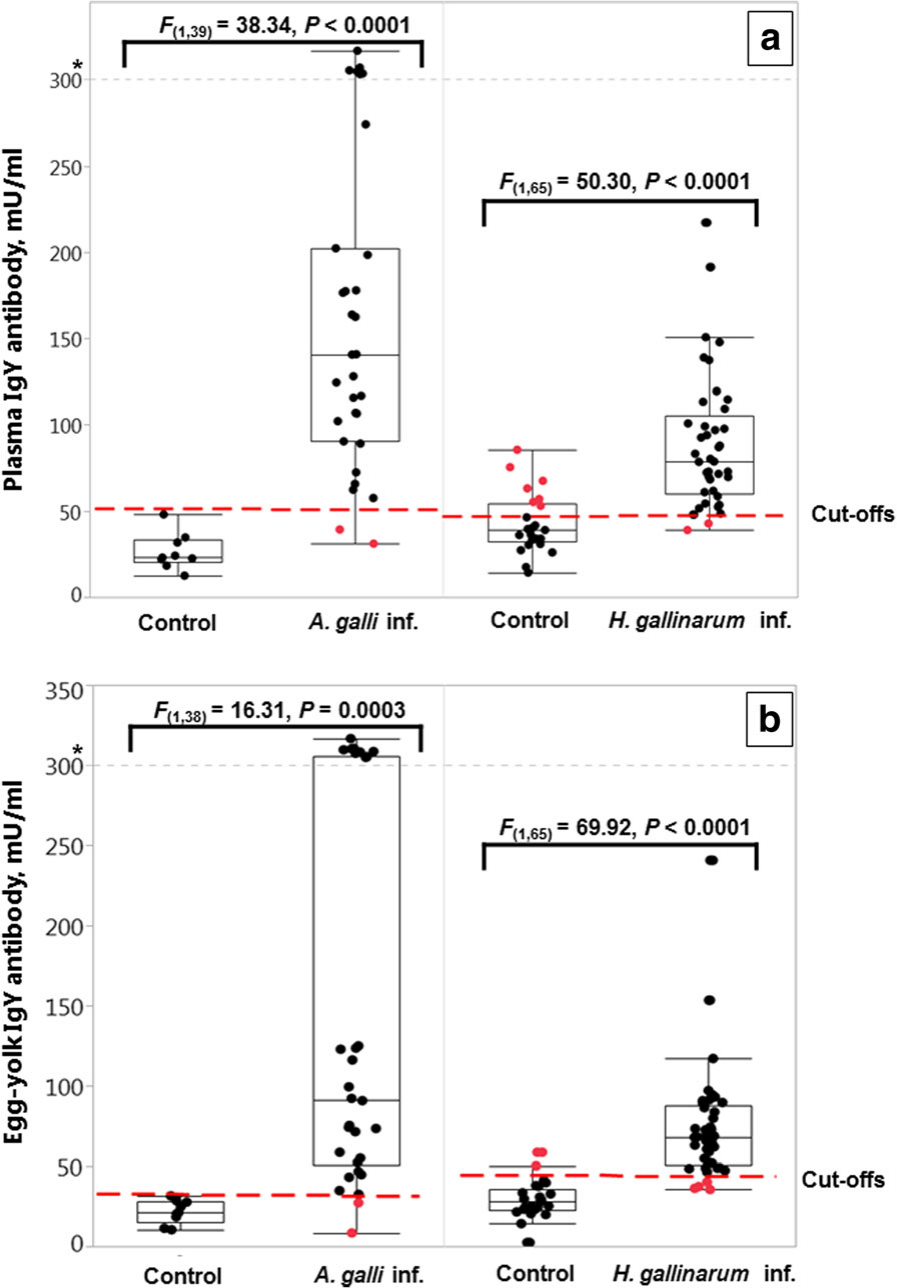
Plasma (**a**) and egg-yolk (**b**) IgY concentrations in uninfected or experimentally infected birds in two experiments. Infected birds received an oral inoculation of 1,000 embryonated eggs of *A. galli* or *H. gallinarum* and necropsied 28 or 30 week p.i., respectively. Data are shown with the outlier box plots with the vertical line within the box representing the sample median, and the lower and upper end of the box representing the 25^th^ and 75^th^ quantiles, respectively. Each dot is an independent observation. The extent of the whiskers is calculated as 25^th^ and 75^th^ quartiles plus or minus 1.5 × interquartile range, respectively. Outliers above the grey line (* > 300) are shown on an extended reduced scale [reduced y-axis = (y*0.01 + 300)] to depict a focused picture of the whole data. The dashed red lines indicate the cut-off values quantified by the ROC analyses for each nematode and sample matrix (see Table 2 for details). The figure represents raw data, but the statistical comparisons are based on the log-transformed data

The assay quantified large differences in plasma or in egg yolk IgY levels between infected and uninfected animals in two experiments, each performed with either of the nematode species. Overall average plasma and egg-yolk antibody concentrations of the uninfected and infected birds in each experiment are summarized in Additional file 3: Table S1. As shown in Fig. 1a, there was a significant effect of *A. galli* infection on *A. galli*-specific antibody concentrations in plasma samples (*F*_(1,39)_ = 38.34, *P* < 0.0001). Similarly, *H. gallinarum* infected birds had higher (*F*_(1,65)_ = 50.30, *P* < 0.0001) antibody levels as compared to their age-matched uninfected controls. Similar to plasma samples, both *A. galli* (*F*_(1,38)_ = 16.31, *P* = 0.0003) and *H. gallinarum* (*F*_(1,65)_ = 69.92, *P* < 0.0001) mono-infections resulted in an elevated level of antibody concentration in the egg-yolks of the infected birds (Fig. 1b).

### Correlations between worm burden and infection proxies

The total worm burden did not significantly correlate with plasma or egg yolk antibody concentrations of the chickens infected with either of the nematodes (*P* > 0.05). There was a moderate-high positive correlation (*r*_(31)_ = 0.42, *P* = 0.0191) between EPG and total worm burden in experimentally *A. galli*-infected chickens, whereas the correlation between EPG and worm burden for *H. gallinarum* was not significantly different from zero (*r*_(41)_ = -0.14, *P =* 0.3847).

### ROC curve analyses

**Fig. 2:**
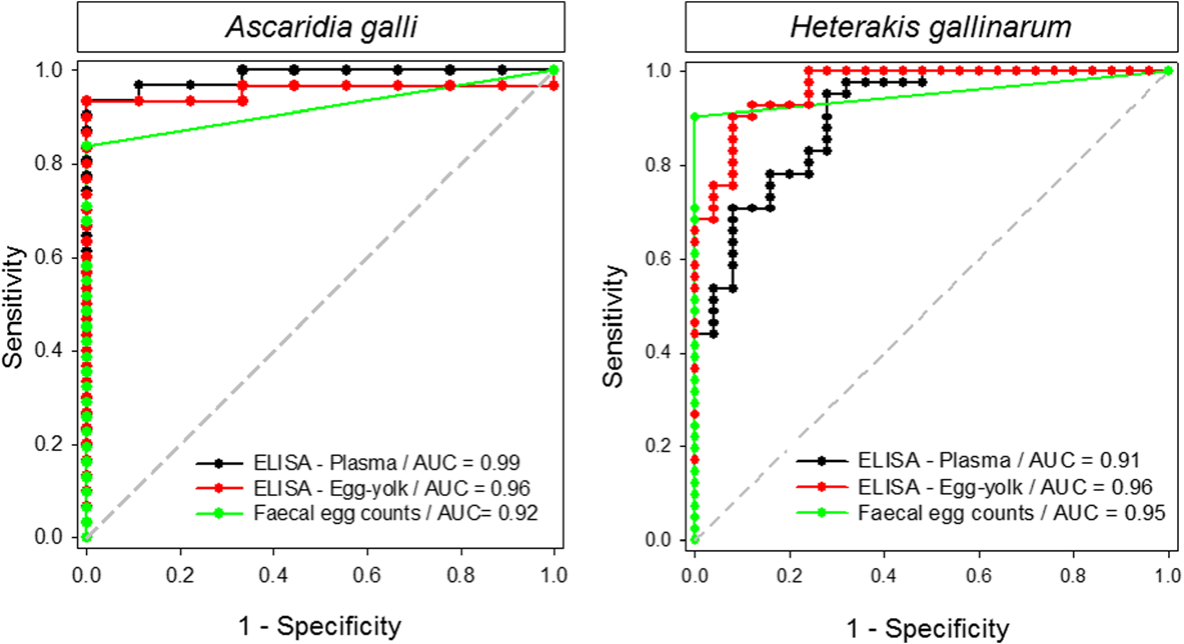
Overall diagnostic accuracy of the ELISA using plasma or egg-yolk samples in comparison to FECs. Area under the ROC curves (AUC) for the ELISA assays based on plasma antibody, egg yolk antibody or for faecal egg counts in chickens experimentally infected with *A. galli* or *H. gallinarum*. The dashed diagonal line corresponds to the half of the maximum AUC (1.0). The farther the location of the ROC curve is from the diagonal line, the higher the total test accuracy

The area under the ROC curve (AUC ± SE), a measure of total test accuracy, indicated that the assay performed with high accuracy (AUC > 0.90) for both nematodes using either plasma or egg yolks (Fig. 2 and Table 2). Total test accuracy of the assay with plasma samples (AUC = 0.99 ± 0.014) tended to be higher (*χ* ^2^ = 3.457, *df* = 1, *P =* 0.0630) than FEC (AUC = 0.92 ± 0.033) for *A. galli* (Table 2). Sensitivity of the assay was 94 and 93% with plasma and egg yolk samples, respectively, whereas FEC yielded in a sensitivity of 84% in *A. galli* experiment. Both the ELISA and FEC identified true negative animals with the highest possible specificity (100%) in the *A. galli* experiment.

**Table 2:** Diagnostic ability of the ELISA using plasma or egg yolk samples in comparison to faecal egg counts (FEC) obtained from 24 h faeces in chickens with mono-*Ascaridia galli* or -*Heterakis gallinarum* infections

Worm species	Parameter	ELISA - Plasma	ELISA - Egg yolk	FEC
*A. galli*	AUC ± SE	0.99 ± 0.014^a^	0.96 ± 0.034	0.92 ± 0.033^a^
	Cut-off	52.9 mU/ml	32.0 mU/ml	25 EPG
	Sensitivity, % (95% CI)	94 (79–99)	93 (78–99)	84 (66–95)
	Specificity, % (95% CI)	100 (66–100)	100 (66–100)	100 (66–100)
*H. gallinarum*	AUC ± SE	0.91 ± 0.037^b^	0.96 ± 0.019^b^	0.95 ± 0.023
	Cut-off	47.2 mU/ml	43.8 mU/ml	25 EPG
	Sensitivity, % (95% CI)	95 (83–99)	90 (77–97)	90 (77–97)
	Specificity, % (95% CI)	72 (51–88)	92 (74–99)	100 (86–100)

^a^Area under the ROC curves (AUC) sharing the superscript tend to differ between corresponding tests in *A. galli* infection (Chi-square test: *χ* ^2^ = 3.457, *df* = 1, *P* = 0.0630)

^b^AUC sharing the superscript tend to differ between corresponding tests in *H. gallinarum* infection (Chi-square test: *χ* ^2^ = 2.921, *df* = 1, *P* = 0.0874)

Although the assay tended (*χ* ^2^ = 2.921, *df* = 1, *P =* 0.0874) to perform better with egg-yolks (AUC = 0.96 ± 0.019) than with plasma (AUC = 0.91 ± 0.037) in *H. gallinarum* experiment, no significant difference was quantified when the assay with either sample type was compared with FEC (AUC = 0.95 ± 0.023). Sensitivity of the assay was 95 and 90% with plasma and egg yolk samples, respectively whereas FEC derived from 24 h faeces samples provided a sensitivity of 90% (CI: 77–97%). None of the negative faeces samples was found to be positive with FEC, whereas ELISA classified falsely 8 and 28% of uninfected controls as *H. gallinarum*-infected by using egg-yolks and plasma samples, respectively (Fig. 1 and Table 2).

### Seroconversion

**Fig. 3:**
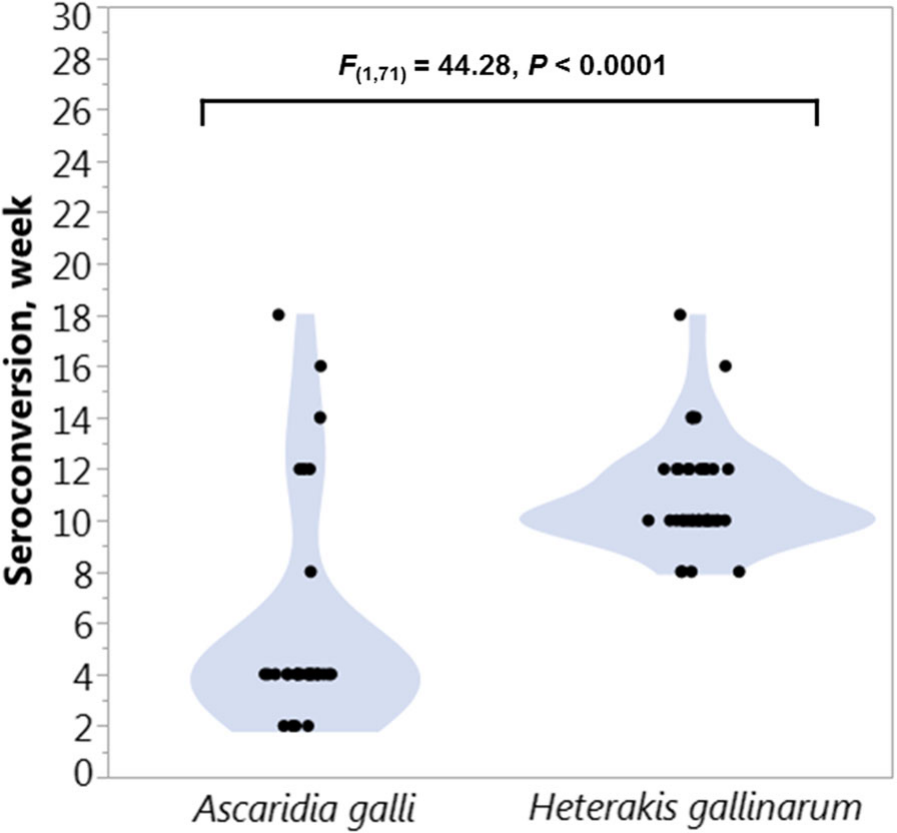
Seroconversion with plasma IgY and worm specific cut-off values. Contour-plots showing seroconversion for experimentally *A. galli*- or *H. gallinarum*-infected chickens using plasma samples and worm specific cut-off values for plasma samples (52.9 and 47.2 mU/ml for *A. galli* and *H. gallinarum*, respectively). Notice that the Y-axis is scaled to the duration of the *H. gallinarum* experiment (30 week). For the *A. galli* experiment, the duration was 28 weeks

As shown in Fig. 3, the time required to quantify a sufficient level of antibodies for the first time (seroconversion) in plasma samples with the species-specific cut-off values (see Table 2) was significantly (*F*_(1,71)_ = 44.28, *P* < 0.0001) lower for *A. galli* infected animals (5.8 ± 0.77 weeks) than for *H. gallinarum* infected animals (10.9 ± 0.32 weeks). Plasma antibody levels of more than 80% of *A. galli-*infected birds exceeded the cut-off values already by the 4^th^ week of infection. *H. gallinarum*-infected birds showed seroconversion for the first time by the 8^th^ week of infection. All the birds infected with either nematode were positively (> cut-off) identified latest by 18 weeks p.i. (Fig. 3).

### Validation of the ELISA with naturally occurring mixed infections

**Fig. 4:**
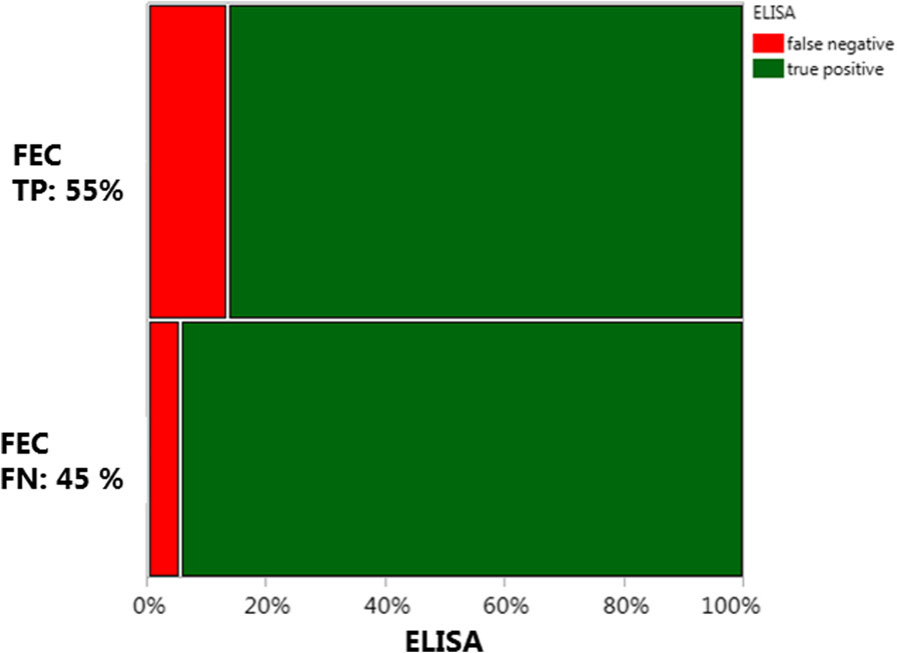
Qualitative comparison of the ELISA with FECs using naturally infected animals from the field. A mosaic plot of the field data representing two-way percentages of false negative (FN) and true positive (TP) cases for naturally infected hens identified with faecal egg counts (FEC) and ELISA

**Fig. 5:**
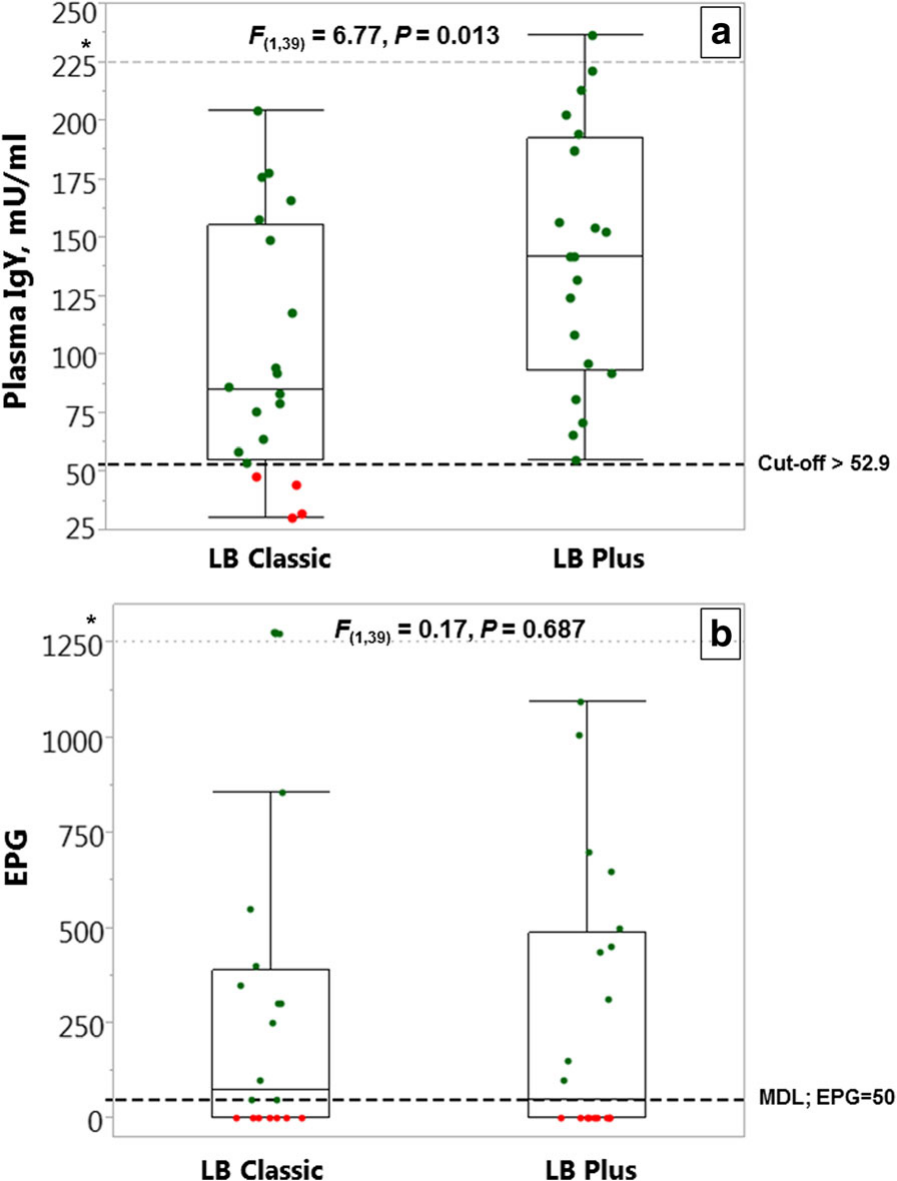
Plasma antibody (**a**) and FECs (**b**) in naturally infected chickens from the field. Plasma samples (*n* = 40) were obtained from chickens with naturally occurring nematode infections that were used in a previous study exploring host genetic resistance to nematodes [4]. The dashed line, indicating the cut-off value (52.9 mU/ml), was determined by the ROC analyses for experimentally *A. galli* infected animals and used here as the threshold to classify the hens as infected or uninfected. Following this separation, the green () and red () dots represent true and false positive cases, respectively. Outliers above the dashed grey line (* > 300) are shown on an extended reduced scale [reduced y-axis = (y*0.01 + 300)] to depict a focused picture of the whole data. The FECs (faecal egg counts) are expressed as EPG (number of eggs per gram faeces). MDL: minimum detection limit of the McMaster egg counting technique (EPG = 50). For the properties of the box plots, see the explanations given in Fig. 1. The figure represents raw data, but the statistical comparisons are based on the log-transformed data

All the naturally infected hens harboured both *H. gallinarum* and *A. galli,* whereas 86% of the birds were also infected with *Capillaria* spp. (Table 3). On average (Mean ± SE) each hen harboured 240 ± 41.8 worms with a high variation ranging from 17 to 1,451 worms/hen. Percentages of true positive animals using the cut-off value (52.9 mU/ml) that was determined by the ROC analyses for experimentally *A. galli* infected animals were 90% across two genotypes (Fig. 4). Out of 40 naturally infected hens from which both faeces and plasma samples were obtained, 18 (45%) were negative with FEC (Fig. 4). Overall average plasma antibody concentration of the naturally infected hens was 120 ± 9.1 (Table 3). Although the plasma antibody concentration was higher (*F*_(1,39)_ = 6.77, *P* = 0.013; Fig. 5a) in LB Plus than in LB Classic hens, there was no significant difference (*F*_(1,39)_ = 0.17, *P* = 0.687; Fig. 5b) in faecal egg concentrations (EPG) between the two genotypes. Average EPG in the naturally infected hens across the two genotypes was 279 ± 60 (Table 3). Total worm burdens of the two genotypes did not differ significantly (*F*_(1,39)_ = 0.08, *P* = 0.774).

**Table 3:** Descriptive statistics for worm burdens, faecal egg counts and plasma antibody concentrations of field chickens^a^ naturally infected with gastrointestinal nematodes

Item	Mean	SE	Min	Max	Prevalence (%)^b^
*H. gallinarum* (*n*/bird)	192	40.9	3	1408	100
*A. galli* (*n*/bird)	36	6.0	1	126	100
*Capillaria* spp. (*n*/bird)	13	2.2	0	52	86
Total worm burden (*n*/bird)^c^	240	41.8	17	1451	100
EPG	279	60.0	0	1278	55
Plasma antibody (mU/ml)	120	9.1	30.6	236.6	90

^a^The chickens (*n* = 40) were brown laying hens of two closely related genotypes, kept on an organic farm and necropsied in the end of laying period at an age of  ≥ 79 weeks

^b^Percentage of chickens infected with each or any nematode species. For EPG it indicates percentage of chickens with EPG positive samples (e.g. EPG ≥ 50). For plasma antibody it indicates percentage of animals correctly classified as infected using the cut-off (> 52.9 mU/ml)

^c^Calculated as the sum of all worms from three nematode species. For calculation of prevalence, the existence of at least one species was considered to be positive

*Abbreviations*: *Min* minimum, *Max* maximum, *SE* standard error

## Discussion

The two hypotheses examined in the present study could not be rejected. It was shown that the ELISA based on somatic antigens of *A. galli* not only identified with high accuracy animals infected with this nematode species but also those infected with *H. gallinarum*, indicating the production of cross-reacting antibodies in chickens against both worm species. The ELISA provided a higher diagnostic test accuracy, particularly for naturally infected animals from the field, compared with the FECs. The results also demonstrated that the IgY antibodies found in egg yolks are as informative as plasma samples; therefore, host friendly, non-invasive sampling is possible. Nevertheless, the quantitative informative ability of the assay is limited compared with FECs, which show a higher correlation with the actual *A. galli* infection intensity (e.g. *A. galli* burden) of the birds.

### Intensity of infections and comparability with natural ones

Despite the importance of re-emerging nematode infections in chickens, diagnostic tools for these infections are mainly limited to coprological analyses with relatively low accuracy [3] and precision [22]. Although the pig industry is facing a similar infection threat from roundworms, particularly *Ascaris suum*, a successful assay has been recently developed to identify infected animals [40, 41]. The data reported in the experimental part of this study were derived from animals with a long-history (≥ 28 weeks) of infection. The life-cycle of *H. gallinarum* and *A. galli* can be completed as early as 5 and 8 weeks, respectively, with both nematodes requiring a similar period of time for their eggs to become fully embryonated [42, 43], although *A. galli* has a longer prepatent period [11, 34]. Both *A. galli* and *H. gallinarum* infections were patent and included re-infections, as indicated by the presence of larvae that accounted for approximately 15 and 22% of *H. gallinarum* and *A. galli* populations, respectively. Average worm burdens with either parasite were higher than reported in our previous experimental infection studies [11, 13, 34], most likely due to the longer period of time in this study, allowing re-infections to occur continuously. However, the *per capita* worm fecundity (number of eggs excreted per female within a day) was much lower (44 and 76% for *H. gallinarum* and *A. galli*, respectively) than previously reported for both species in chickens with lower infection intensities [21, 34], indicating a strong density-dependency in worm fecundity from crowded populations. Infection intensities resulting from the mono-species inoculations were indeed even higher than the levels quantified in the field study. As re-infections must have occurred continuously, the higher worm burdens with both species not only indicate the effectiveness of the oral-faecal transmission route of the infections but probably also point to the lack of an effective acquired immunity against both nematodes. Because the intensities of infections resulting from the experimental inoculation of eggs and/or naturally occurring re-infections were even higher than the levels quantified in the field study, and the worm populations consisted of both mature and juvenile stages, we infer from the results that the experiments were highly comparable to those occurring under natural conditions, with the exception that all the birds were exposed to infections with mono-species. The mono-species infections enabled us to compare the diagnostic accuracy of the ELISA for both worm species using plasma and egg yolk samples in comparison to a commonly used faecal egg counting technique (e.g. McMaster technique).

### Diagnostic ability of the ELISA

The total diagnostic test accuracy of the ELISA, as quantified with area under curve of the ROC analyses, was high for both nematodes using plasma or egg yolks as sample matrix (AUC > 0.90). Although accuracy of ELISA with plasma samples (AUC = 0.99) tended to be higher than that of FECs (AUC = 0.92) in *A. galli* experiment, there was no significant difference in *H. gallinarum* experiment. It is of crucial importance to note that the faecal samples collected in the infection experiments were not randomly taken, as their informative ability would otherwise be strongly limited due to diurnal fluctuations in egg excretion of the nematodes [21] and due to faeces-related factors [13, 22]. They were well-mixed samples that were derived from 24 h faeces, which high probably prevented a strong underestimation of the test accuracy by FECs, particularly for *H. gallinarum,* as was the case for naturally multi-species-infected animals from the field. Whilst 90% of the naturally infected hens were correctly identified with the ELISA, 45% of the infected hens tested (false) negative with the FEC (Fig. 4). Both the ELISA and FEC tests were able to identify true negative animals with the highest possible specificity (100%) in the *A. galli*-experiment, whereas 8–28% of uninfected control birds were falsely classified with ELISA in the *H. gallinarum* experiment, altough no significant difference was quantified in total test accuracy (AUC) between ELISA and FEC. The specificity of the ELISA with plasma samples (72%) was somewhat lower than with egg yolks (92%) for *H. gallinarum.* The high specificities of FECs (100%) are explained by the fact that nematode eggs can only be found in faeces of infected animals harbouring mature/fecund female worms, although false positive cases have been reported for uninfected pigs practicing coprophagy [44]. The diagnostic success of the ELISA in the field will not depend only on high test sensitivity and specificity to distinguish correctly between infected and non-infected animals, but also on the true prevalence of the nematode infections, which are extremely high [1–5]. This is because of the fact that the likelihood of false-positive and false-negative testing classification errors is strongly influenced by the true prevalence of infections [45]. Consequently, an assay with high specificity and sensitivity would not be that valuable if true prevalence of an infection is low, mainly because positive predictive value (i.e. probability of infection given a positive test result) decreases as prevalence decreases [45].

*Ascaridia galli-*infected animals required a shorter seroconversion time (5.8 weeks) than *H. gallinarum-*infected animals (10.9 weeks). Most of *A. galli*-infected animals (> 80%) were classified correctly as infected already by the second sampling time (e.g. 4 weeks p.i.) indicating the potential of the assay to be used for diagnosis of early-stage *A. galli* infections, which cannot be determined by faecal egg counts as it takes 5 to 8 weeks for the infection to become patent [46].

The difference in seroconversion time may at least partly result from the age of chickens used for the experimental infections. *H. gallinarum* infected birds were younger than *A. galli*-infected birds. The younger age of the birds may be associated with the lower IgY antibody production, as is known for chickens [47]. *Ascaridia galli* is known to have a mucosal/histotrophic phase up to 54 days [48], which is considered to be a normal part of its life-cycle [49]. In contrast, *H. gallinarum* has a very short tissue-associated phase maximum up to 12 days [50]. As the tissue-embedded larvae may induce a higher antigenic stimulation [25], the shorter seroconversion time for *A. galli* may also partly be due to presence and duration of the mucosal/histotrophic phase. The seroconversion time might have been influenced not only by the mucosal/histotrophic phase and age of animals but also by level of infections as well as by the number of continuously occurring exposures or re-infections.

### One assay for diagnosis of two nematodes

The two prototype assays, each developed for diagnosis of either *A. galli* or *H. gallinarum,* showed good agreement (see Additional file 2: Figure S2). The presence of antibodies that could be detected by either assay was a strong indication that chickens develop similar, if not the same, antibodies that cross-react to both nematode species. The molecular weight of antigens involved in the reactivity against both embryonated egg antigens and against adult *A. galli* antigens have been described by Marcos-Atxutegi et al. [25]. According to these authors, *A. galli* antigens responsible for the stimulation of specific IgY are polypeptide molecules of 30–34, 44–55 and 58–90 kDa. These results are in agreement with our data for *A. galli* somatic antigens (Additional file 1: Figure S1b) and are supported also by the data of [51] who observed antigenic bands with molecular weights of 55–72 kDa. By using a solubilisation protocol, we were able to increase the number of potential antigens, especially in the range of 40–220 kDa, to increase the spectrum of measurable worm antibodies, resulting in fewer false negative results. Although the chemical structures of these antigens are not fully known, Jordanova et al. [52] described a protein (Ag-Ibp55) with a molecular weight of 55 kDa that represents a novel type of lipid-binding protein and may act as the nematode antigen. Although similar data are not available for *H. gallinarum*, our results indicate that the chicken host does not differentiate between somatic antigens of both nematodes, and produces similar, if not the same, antibodies that can be quantified with the same assay. This implies the non-specificity of the IgY antibodies measured by ELISA for either worm species. Despite the longer seroconversion for *H. gallinarum* than for *A. galli*, the assay can be used generally to identify animals infected with either ascarid, which are involved in naturally occurring infections, mostly together [2, 3, 5]. The similarity in antigenic stimulations induced by *A. galli* and *H. gallinarum* may be related to the phylogenetic closeness of the two species [6, 7].

### Measuring antibodies in egg yolk

IgY is the avian equivalent of mammalian IgG [53]. IgG and IgY are functionally homologous while having fundamental structural differences in their molecules [31, 54]. The transfer of IgY from hen serum via the yolk to the circulation of the embryo is also analogous to cross-placental transmission in mammals with IgG [32]. Although IgA and IgM are mainly found in the albumen, IgY is stored in the egg yolk [32, 54]. Our recent data indicate a very strong correlation (*r* = 0.89) between plasma and egg yolk concentrations of IgY in *A. galli* infected chickens [55]. The objectively determined cut-off values were smaller for egg yolk samples than for plasma samples in both *A. galli* and *H. gallinarum* experiments, indicating smaller minimum detection levels for the egg yolk samples. One of the features of IgY is that their half-life ranges from 36 to 65 h [56], which is substantially shorter than that of IgG in mammalians [54, 57], e.g. 15 days in sheep [58]. The shorter half-life of circulating IgY, which may cause fluctuations in plasma concentrations over time [56], may explain why egg yolks provided a higher test accuracy than the plasma samples for *H. gallinarum*. From an animal welfare point of view, both captivity for the collection of individual faecal samples and puncturing birds for the collection of blood to be used for serological analyses are not favourable. As the egg yolk IgY antibodies provided high test accuracy with both nematodes (AUC = 0.96), and the eggs can be collected from the animals in a non-invasive, host-friendly way, we suggest the ELISA system with egg yolk samples as an accurate diagnostic test for identifying *A. galli and H. gallinarum* infected chickens. Because the egg samples were taken at the end of the study period, the transfer time of IgY to egg yolks remains to be clarified in further studies.

### Quantitative infection proxies

Although there were multiple-fold and statistically significant differences in the quantity of antibody levels (plasma and egg yolk) between infected and uninfected animals (depending on parameter and worm species, on average 2 to 14 times higher), the ELISA system has not yet fully been proven to function quantitatively. For this, studies including sentinel birds necropsied at regular intervals to monitor changes in the relationship between worm burden with changing demographic composition (e.g. larval stages and first generation mature worms resulting from experimental infections and the mixed stage-populations due to re-infections) and antibody levels are required.

Among the three tests, FECs correlated better with worm burdens than the ELISA with plasma or egg yolks for both nematodes in *A. galli* experiment. In *H. gallinarum* experiment, however, there was no significant correlation between worm burden and FEC, indicating inadequacy of FECs to quantify infection intensity for this nematode even if based on well-mixed 24 h faecal samples. Although the FECs provided relatively high qualitative-diagnostic information (sensitivity 90%), no reliable quantitative information on the infection intensity of *H. gallinarum* could be derived by FECs. This might at least partly be related to the impaired fecundity of female worms in crowded worm populations as discussed earlier. Because it is known that both inverse- and density dependency mechanisms regulate fecundity of *H. gallinarum* [34], the expected linear relationship between worm burden and FECs might have simply been masked as a consequence of the existence of different infra-populations under influence of either inverse-density dependency or density dependency effects in the entire worm population.

The results reported for correlation analysis included data only from experimentally mono-species infected animals. To mimic infections under natural conditions, where infected and uninfected animals are present in the same population, we additionally pooled data from two stratified groups (i.e. infected and uninfected animals) for correlation analysis to check whether the infection proxies (i.e. plasma and egg yolk antibody and EPG) would correlate with worm burden. This is somewhat against the statistical rationale behind data pooling but fits the natural conditions. In this way, we estimated positive correlations between *A. galli* counts and antibody concentrations for plasma (*r*_(40)_ = 0.65, *P* < 0.0001) and egg yolks (*r*_(39)_ = 0.60, *P* < 0.0001), respectively (Additional file 4: Figure S3). The corresponding correlations for *H. gallinarum* were highly positive (*r*_(66)_ = 0.67, *P* < 0.0001 and *r*_(66)_ = 0.73, *P* < 0.0001, respectively). Following this scenario, correlations between EPG and worm burden also improved for both nematodes, i.e. *r*_(40)_ =0.73 (*P* < 0.0001) and *r*_(66)_ = 0.83 (*P* < 0.0001) for *A. galli* and *H. gallinarum*, respectively. However, as the infection intensity estimates should only apply to infected animals (e.g. animals with worm burden ≥ 1), the correlations including uninfected control birds are, probably, of limited practical use.

## Conclusion

We conclude that antibodies developed against *A. galli* can successfully be used to identify both *A. galli*- and *H. gallinarum*-infected animals with the newly developed ELISA system. Antibodies accumulated in egg yolks are as informative as plasma samples; therefore, a host friendly, non-invasive sampling is possible. Although the assay with plasma samples reveals qualitative information of higher quality than the faecal egg counts on the infection status of naturally infected birds, the latter is still a better tool to assess the intensity of *A. galli* but not of *H. gallinarum* infections in chickens.

## Additional files

Additional file 1: Figure S1. **a** The ELISA system based on enzyme-conjugated secondary antibody against chicken IgG (IgY) bound to micro-wells coated with the *A. galli* antigen. **b** Analysis of *A. galli* proteins with potential antigenic properties in both soluble proteins and in solubilized pellet extracts by electrophoresis (SDS-PAGE analysis). For the ELISA, both fractions were pooled to increase antibody-detectable antigens. (TIF 136 kb)

Additional file 2: Figure S2. Plasma antibody concentrations (mU/ml) in chickens experimentally infected (red dot) with *A. galli* (A) or *H. gallinarum* (B) or from uninfected control birds (green dot). Plasma samples were analysed both with the worm-specific ELISA and with the alternative assay developed for the detection of antibodies against the other worm species (*A. galli vs H. gallinarum* or *vice versa*). Antibody concentrations measured with the species-specific assay are shown on the Y-axes, whereas results with the alternative assay are shown on the X-axes of the corresponding plots. The fitted lines (dark blue line) are shown together with the confidence regions. Number of observations: **a** Control (*n* = 8); Infected (*n* = 37); **b** Control (*n* = 19); Infected (*n* = 20). The figure represents raw data, but the statistical comparisons are based on the log-transformed data. (TIF 111 kb)

Additional file 3: Table S1. Average (Mean ± SE) plasma and egg-yolk antibody concentrations of infected birds and their uninfected-control counterparts in *A. galli* and *H. gallinarum* experiments. (DOC 29 kb)

Additional file 4: Figure S3. Relationships between worm burdens and infection proxies based on pooled data. Linear relationships between worm burdens with plasma antibody, egg yolk antibody and faecal egg counts in chickens experimentally infected (red dot) with *Ascaridia galli* or with *Heterakis gallinarum* and uninfected control birds (green dot). Note that the correlations are based on pooled data from infected and uninfected controls within each nematode infection. (TIF 161 kb)

## Abbreviations

AUC: area under curve
ELISA: Enzyme-Linked Immunosorbent Assay
EPG: number of eggs per gram of faeces
FEC: faecal egg counts
MDL: minimum detection level
ROC: Receiver Operating Characteristic
