# Supplementary figures and images for: A comprehensive evaluation of an ELISA for the diagnosis of the two most common ascarids in chickens using plasma or egg yolks
Source: Parasit Vectors. 2017 Apr 18;10:187. doi: 10.1186/s13071-017-2121-9 (PMC5395908; doi:10.1186/s13071-017-2121-9)

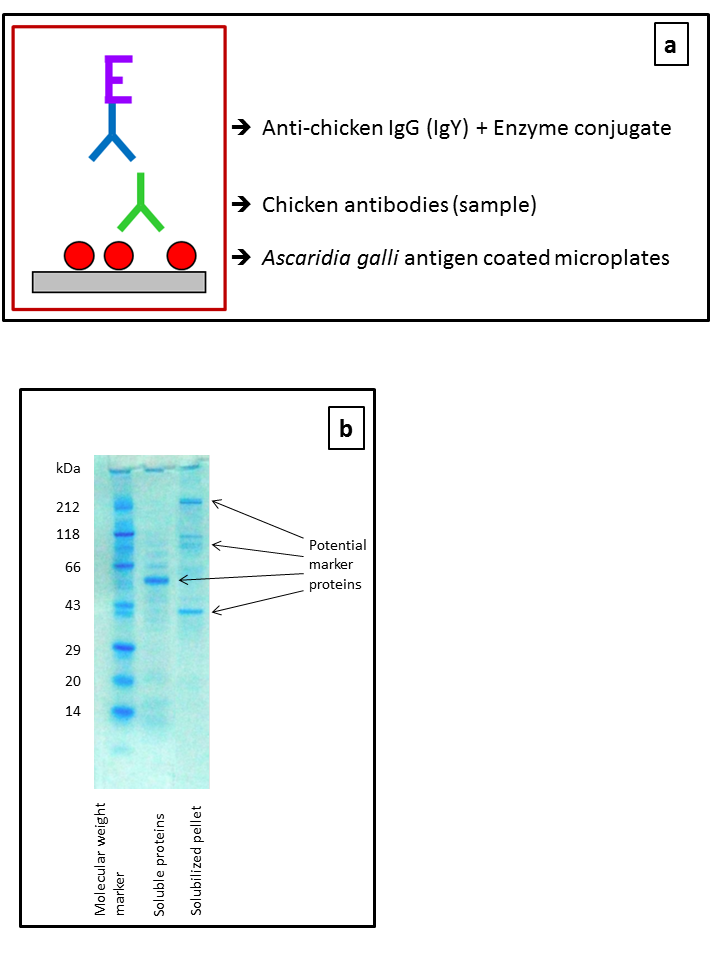

Supplement: Supplementary file 1 — a The ELISA system based on enzyme-conjugated secondary antibody against chicken IgG (IgY) bound to micro-wells coated with the A. galli antigen. b Analysis of A. galli proteins with potential antigenic properties in both soluble proteins and in solubilized pellet extracts by electrophoresis (SDS-PAGE analysis). For the ELISA, both fractions were pooled to increase antibody-detectable antigens. (TIF 136 kb) [file 13071_2017_2121_MOESM1_ESM.tif]

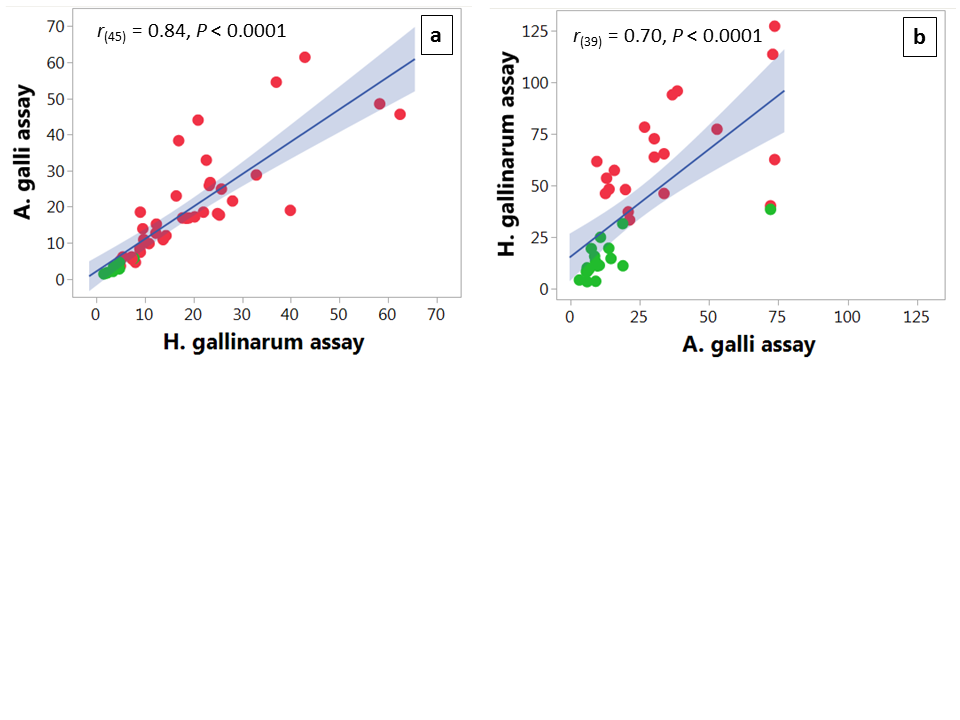

Supplement: Supplementary file 2 — Plasma antibody concentrations (mU/ml) in chickens experimentally infected (red dot) with A. galli (A) or H. gallinarum (B) or from uninfected control birds (green dot). Plasma samples were analysed both with the worm-specific ELISA and with the alternative assay developed for the detection of antibodies against the other worm species (A. galli vs H. gallinarum or vice versa). Antibody concentrations measured with the species-specific assay are shown on the Y-axes, whereas results with the alternative assay are shown on the X-axes of the corresponding plots. The fitted lines (dark blue line) are shown together with the confidence regions. Number of observations: a Control (n = 8); Infected (n = 37); b Control (n = 19); Infected (n = 20). The figure represents raw data, but the statistical comparisons are based on the log-transformed data. (TIF 111 kb) [file 13071_2017_2121_MOESM2_ESM.tif]

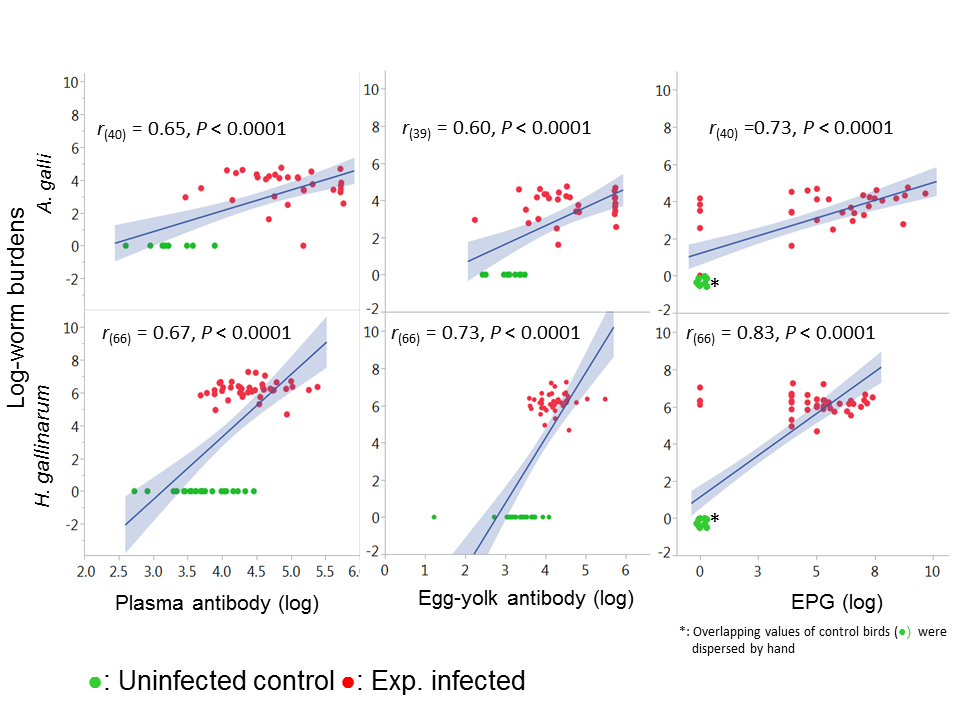

Supplement: Supplementary file 4 — Relationships between worm burdens and infection proxies based on pooled data. Linear relationships between worm burdens with plasma antibody, egg yolk antibody and faecal egg counts in chickens experimentally infected (red dot) with Ascaridia galli or with Heterakis gallinarum and uninfected control birds (green dot). Note that the correlations are based on pooled data from infected and uninfected controls within each nematode infection. (TIF 161 kb) [file 13071_2017_2121_MOESM4_ESM.tif]
