# Supplementary material for: A comprehensive evaluation of an ELISA for the diagnosis of the two most common ascarids in chickens using plasma or egg yolks
Source: Parasit Vectors. 2017 Apr 18;10:187. doi: 10.1186/s13071-017-2121-9 (PMC5395908; doi:10.1186/s13071-017-2121-9)
Supplement: Supplementary file 3 — Average (Mean ± SE) plasma and egg-yolk antibody concentrations of infected birds and their uninfected-control counterparts in A. galli and H. gallinarum experiments. (DOC 29 kb) [file 13071_2017_2121_MOESM3_ESM.doc]

**Additional file 3: Table S1**

Average (Mean ± SE) plasma and egg-yolk antibody concentrations of infected birds and their uninfected-control counterparts in *A. galli* and *H. gallinarum* experiments

| **Item** | ***A. galli*** | | ***H. gallinarum*** | |
| --- | --- | --- | --- | --- |
|  | **Control**  **(n = 9)** | **Infected**  **(n = 31*)** | **Control**  **(n = 25)** | **Infected**  **(n=41)** |
| Plasma antibody, mU/mL | 26 ± 3.5 | 231 ± 55.2 | 42.3 ± 3.4 | 89 ± 6.1 |
| Egg-yolk antibody, mU/mL | 22 ± 2.5 | 316 ± 77.6 | 28.9 ± 2.3 | 73.4 ± 5.5 |

*: n= 30 for egg yolk antibody
